# Modelling the return on investment of preventively vaccinating healthcare workers against pertussis

**DOI:** 10.1186/s12879-015-0800-8

**Published:** 2015-02-19

**Authors:** Luqman Tariq, Marie-Josée J Mangen, Anke Hövels, Gerard Frijstein, Hero de Boer

**Affiliations:** Division of Pharmacoepidemiology & Clinical Pharmacology, Utrecht Institute for Pharmaceutical Sciences, Utrecht University, Utrecht, The Netherlands; GlaxoSmithKline BV, Zeist, The Netherlands; Julius Center for Health Sciences and Primary Care, University Medical Center Utrecht, Utrecht, The Netherlands; Academic Medical Centre Amsterdam, Meibergdreef 9, 1105 AZ Amsterdam, The Netherlands

**Keywords:** Pertussis, Nosocomial outbreak, Healthcare workers, Return on investment, Vaccination, Cost

## Abstract

**Background:**

Healthcare workers (HCWs) are at particular risk of acquiring pertussis and transmitting the infection to high-risk susceptible patients and colleagues. In this paper, the return on investment (ROI) of preventively vaccinating HCWs against pertussis to prevent nosocomial pertussis outbreaks is estimated using a hospital ward perspective, presuming an outbreak occurs once in 10 years.

**Methods:**

Data on the pertussis outbreak on the neonatology ward in 2004 in the Academic Medical Center Amsterdam (The Netherlands) was used to calculate control costs and other outbreak related costs. The study population was: neonatology ward staff members (n = 133), parents (n = 40), neonates (n = 20), and newborns transferred to other hospitals (n = 23). ROI is presented as the amount of Euros saved in averting outbreaks by investing one Euro in preventively vaccinating HCWs. Sensitivity analysis was performed to study the robustness of the ROI. Results are presented at 2012 price level.

**Results:**

Total nosocomial pertussis outbreak costs were €48,682. Direct control costs (i.e. antibiotic therapy, laboratory investigation and outbreak management control) were €11,464. Other outbreak related costs (i.e. sick leave of HCWs; restrictions on the neonatology ward, savings due to reduced working force required) accounted for €37,218. Vaccination costs were estimated at €12,208. The ROI of preventively vaccinating HCWs against pertussis was 1:4, meaning 4 Euros could be saved by every Euro invested in vaccinating HCWs to avert outbreaks. ROI was sensitive to a lower vaccine price, considering direct control costs only, average length of stay of neonates on the neonatology ward, length of patient uptake restrictions, assuming no reduced work force due to ward closer and presuming more than one outbreak to occur in 10 years’ time.

**Conclusion:**

From a hospital ward perspective, preventive vaccination of HCWs against pertussis to prevent nosocomial pertussis outbreaks results in a positive ROI, presuming an outbreak occurs once in 10 years.

**Electronic supplementary material:**

The online version of this article (doi:10.1186/s12879-015-0800-8) contains supplementary material, which is available to authorized users.

## Background

Pertussis among healthcare workers (HCWs) is of special concern because of the potential for nosocomial exposure to susceptible patients and other HCWs [1]. HCWs are at particular risk of acquiring pertussis and may transmit the infection to young infants and colleagues [2]. Compared to the general adult population, HCWs are reported to have an almost 1.7-times higher risk of pertussis [3]. In literature, reports of nosocomial pertussis outbreaks following community or hospital exposures of HCWs are available [4-7]. Nosocomial outbreaks not only generate a considerable disease burden in humans, but can also result in substantial control costs and other outbreak related costs for hospitals. The type of expenses include diagnostic testing, provision of antibiotic treatment or prophylaxis, costs associated with furlough of employees, and time spent by occupational health infection control staff to track and identify exposed individuals, as well as costs associated with dissemination of information [2]. Previous studies estimating the nosocomial pertussis outbreak costs among HCWs concluded that these outbreaks resulted in serious adverse health and economic consequences to the hospitals, HCWs, patients and their families [1,5,8,9]. Using a hospital perspective, Ward et al., [5] estimated the total outbreak costs among HCWs at €55,579^1^ for 91 cases in a French hospital. Calugar et al., [1] calculated the total outbreak costs at €76,945^1,2^ for 17 cases in a hospital in the Unites States. From the hospitals’ perspective, Baggett et al., [8] calculated costs of two hospital outbreaks in the United States at €114,526 and €248,998 s^1,2^, respectively. Zivna et al., [9] estimated the total outbreak costs to be €80,428 - €93,088^1,2^ in a tertiary care medical center in the United States. According to Calugar et al., [1] cost savings and benefits can be accrued by vaccinating HCWs against pertussis, with benefits for the hospital estimated at 2.38 times a dollar invested in vaccinating HCWs (USD 2004 estimate). Therefore, prevention of nosocomial pertussis outbreaks by preventively vaccinating HCWs can be beneficial and has the potential to reduce the overall disease and economic burden of pertussis. In the Netherlands, pertussis vaccination was introduced in 1952 with long-established high vaccination coverage of 96-97% [10]. Dutch infants are vaccinated against pertussis on the age of 2, 3, 4, 11 months, and 4 years in the National Immunization Program (NIP) [11], meaning in the first four months, infants are not fully protected against pertussis and disease occurs frequently, especially in years of high pertussis circulation (i.e. every 2–4 years) [10]. A national vaccination recommendation of HCWs has yet to be made in the Netherlands. Nosocomial pertussis outbreaks have occurred in the Netherlands in the past decade [12,13]. However, economic consequences of such pertussis outbreaks and the potential benefits of preventively vaccinating HCWs have not been evaluated. In this paper, we aim to calculate the return on investment (ROI) of preventively vaccinating HCWs against pertussis to prevent nosocomial pertussis outbreaks in a neonatology ward using a hospital ward perspective. Data on the nosocomial pertussis outbreak on the neonatology ward in the Academic Medical Center Amsterdam (AMC) in The Netherlands in the year 2004 (for details see Box A.1 in Additional file 1) were used as a case study to examine the economic impact of a pertussis outbreak in a neonatology ward.

## Methods

### Data collection & study population

During the outbreak period, data were collected by the occupational health service department of the AMC (hereafter referred as “AMC database”) on all control measures undertaken related to newborns, their parents, staff members, and to the organization within the hospital. The study population consisted of: neonatology ward staff members (15 neonatologists, 100 nurses, 18 assistants), parents of newborns (20 fathers, 13 lactating mothers, 7 non-lactating mothers), neonates (20 infants) and parents of 23 newborns who were transferred to another hospital. As data from on the pertussis outbreak was used in an aggregated way without identifying the individual participant, no written informed consent and ethical approval were required from participants to perform the data analysis.

### Assumptions

The following assumptions were made in this study as the AMC database did not capture all data on the outbreak:

- Due to patient uptake restrictions (i.e. for a period of 10 days, no new patients were allowed to be admitted on the neonatology ward), we assumed that the following activities were performed during regular working hours (i.e. not resulting in additional costs for the hospital ward):
  - telephone calls made to parents whose children were transferred to another hospital;
  - time spent by the neonatologist working on controlling the outbreak;
  - survey performed by the occupational health service department as this is normal procedure during an outbreak in the AMC;
  - all drug and vaccination administrations;
  - PCRs done on nasopharyngeal swabs and blood samples taken for serology;
- Based on average Dutch working population [14] we assumed that an average working week of staff members other than neonatologists consisted of 32 hours; neonatologists were assumed to work 42 hours/week [15];
- No further transmission of the pertussis infection took place after restrictions on the patient uptake on the neonatology ward were lifted;
- Reduced work force was required to run the neonatology ward during the period of restrictions on the patient uptake. We assumed that on day 1, 2, 3 and 4 0%, 5%, 10% and 15% reduced work force was required, respectively. On day five and onwards, this assumption was set at 20%.

### Cost estimations

Total outbreak costs were calculated by considering:

- direct control costs (i.e. (i) medical consumption costs containing antibiotics, (ii) laboratory investigation costs, (iii) outbreak control management costs) and
- other outbreak related costs (i.e. (iv) replacing costs for sick hospital staff members, (v) losses due to restrictions on patient uptake on the neonatology ward and (vi) savings due to a reduced work force required on the neonatology ward during patient uptake restriction period).

Dutch prices were used to derive medication costs and other resource unit costs [15-18], and where necessary updated to 2012 using Dutch consumer price index (CPI) [14].

Vaccination costs were calculated by considering:

- catch-up vaccination: vaccination of all HCWs (n = 133) one year after the outbreak based on the list price for Infanrix® IPV (diphtheria, tetanus, acellular pertussis and inactivated poliomyelitis) vaccine (€34.50 per vial) [19,20];
- vaccination of newly employed HCWs staff (assumption 10% per year) for a period of 10 years. New HCWs were assumed to be unvaccinated but would be vaccinated upfront when hired at 100% coverage rate;
- booster vaccination to be provided eight years after first vaccination due to the declining vaccine effectiveness [21].

### Return on Investment (ROI)

ROI of preventively vaccinating HCWs was calculated by dividing the return on investment (i.e. averted outbreak costs, using the AMC outbreak costs as proxy) by the cost of the investment (i.e. cumulative vaccination costs including booster vaccination):

$$ \mathrm{Return}\ \mathrm{On}\ \mathrm{Investment} = \left\{\frac{\mathrm{Averted}\ \mathrm{Outbreak}\ \mathrm{Costs}}{\mathrm{Vaccination}\ \mathrm{Costs}}\right\} $$

and it is presented as a ratio: the amount of Euros saved by averting an outbreak times one Euro invested in vaccinating HCWs. All costs are presented in Euro at 2012 price level and without time-discounting. Discounting is applied in sensitivity analysis.

### Model

The analysis was conducted in MS Excel, version 2007 based on the study population and the input parameters displayed in Table 1. The outcome measures were:

**Table 1:** **Study population and input parameters (all costs are expressed in 2012 Euros)**

	**Value**	**Source**
**Study population (n)**		
Fathers	20	*
Lactating mothers	13	*
Non-lactating mothers	7	*
Newborns	20	*
Parents per child	2	*
Average weight newborn (in kg)	2.2	[22]
Staff members	133	*
**Medical consumption**		
Erythromycin cost per vial (solution of 20 mg)	€0.16	[16]
Erythromycin cost per tablet	€0.34	[16]
Azithromycin cost per tablet	€0.53	[16]
**Laboratory investigation**		
Number of PCRs performed:		
Children	20	*
Staff members	24	*
PCR costs per unit	€106.38	[18]
Number of serological tests performed:		
Children	20 27	* *
Staff members		
Serological test cost per unit	€48.96	[23]
**Outbreak control management**		
Crisis meetings in the hospital	5	*
Duration of a crisis meeting (in minutes)	60	*
Personnel present at every crisis meeting:		
Nurses	7	*
Neonatologists	1	*
Assistants	2	*
Amount of surgical masks used during the outbreak period	24	*
Costs per unit surgical mask	€1.22	[24]
**Replacing sick staff members**		
Average working hours of nurses per week	32	[14]
Number of staff members not able to work for three days after performing the PCR test. Assumed they were all nurses	5	*
Number of hours of nurses absence due to the PCR test	68.57	*Calculated*
Number of staff members absent from the neonatology ward for one week. Assumed they were all nurses.	4	*
Number of hours of nurses absence due to illness (i.e. sick leave)	160	*Calculated*
**Restrictions patient uptake**		
Regular occupation of the neonatology ward, patients per day	15	*
Average length of stay of neonates in neonatology ward (in days)	14	* & Personal communication
Average number of patients admitted on the neonatology ward per day	1,071	*Calculated*
Length of patient restriction uptake on the neonatology ward (in days)	10	*
Number of empty bed-days due to ward closure during the restriction period	58.93	*Calculated*
Cost per patient per day due to patient restriction	€798.18	[23]
**Reduced workforce due to patient uptake restrictions**		
Average number of nurses & assistant working/day in the neonatology ward	30	*
Average number of consultant working/day in the neonatology ward	10	*
Average number of neonatologists working/day in the neonatology ward	6	*
Reduced working force due to ward closure:		
On day 1	0%	*Assumed*
On day 2	5%	
On day 3	10%	
On day 4	15%	
On day 5 and onwards	20%	
Reduced working hours due to ward closure		
Nurses	360	*Calculated*
Others	120	
Neonatologists	0	
**Preventive vaccination**		
Infanrix IPV® costs	€34.50	[20]
Staff members vaccinated	133	*
Average number of new personal in neonatology ward /year (in %)	10	*Assumed*
Average number of new personnel in neonatology ward /year (absolute)	13.30	*Calculated*
Booster vaccination after years	8	[21]
**Tariff personnel**		
The costs for the employer are higher than the tariffs paid to the employees, we therefore multiplied the costs per hour by	2.0	*Assumed*
Tariff per hour/nurses	€64.78	[15]
Tariff per hour/neonatologists	€155.04	[15]
Tariff per hour/others	€62.54	[15]

*During the outbreak period, these data were collected by the occupational health service department of the AMC. In this paper we named this information the AMC database.

- total nosocomial pertussis outbreak costs, split up as costs of antibiotics, costs of laboratory investigations, costs of outbreak control management, costs due to work absence of sick staff members, losses due to restrictions on patient uptake and reduced costs (=savings) due to reduced working force;
- ROI of preventively vaccinating HCW assuming one outbreak within 10 years’ time.

### Sensitivity analysis

Univariate and two-way sensitivity analysis was performed on several input parameters to further test the robustness of the outcomes. In Table 2, the scenarios for the sensitivity analysis together with the values of input parameters are displayed. Amongst other variables, the impact of discounting future outbreak costs and vaccination costs on the ROI was estimated, using a discount rate of 4%, according to Dutch health economic guidelines [15]. Also, the number of pertussis outbreaks in a period was varied (i.e. once or twice in 10 years, once in 20 years).

**Table 2:** **Scenarios in the univariate and two-way sensitivity analysis**

**Nr.**	**Scenarios**	**Range for sensitivity analysis**	***Source***
**1**	**Base case**		
**2**	Average working days for nurses per week	3-5	*−1 day and + 1 day*
**3**	Average length of stay of neonates in neonatology ward	7-21	*0.5 and 1.5 × base case*
**4**	Length of patient restriction uptake on the neonatology ward (in days)	5-15	*0.5 and 1.5 × base case*
**5**	Average number of nurses & assistant working/day in the neonatology ward	20-40	*0.5 and 1.5 × base case*
**6**	Average number of consultant working/day in the neonatology ward	5-15	*0.5 and 1.5 × base case*
**7**	Number of staff members not able to work for 3 days after performing the PCR test	0-10	*Assumed*
**8**	Average number of new personnel in neonatology ward /year (in %)	5-15	*0.5 and 1.5 × base case*
**9**	No reduced working hours for nurses, neonatologists and other HCW due to ward closure	0	*Assumed*
**10**	Vaccine price	€18,30	[17]
**11**	Costs considered in the ROI - only direct control costs	€11.464	*Assumed*
**12**	Undiscounted outbreak and vaccination costs with 2 outbreaks in 10 years	0%	*Assumed*
**13**	Discounted outbreak and vaccination costs with 1 outbreak in 10 years	4%	[15]
**14**	Discounted outbreak and vaccination costs with 2 outbreaks in 10 years	4%	[15]
**15**	Undiscounted outbreak and vaccination costs with 1 outbreak in 20 years	0%	*Assumed*
**16**	Discounted outbreak and vaccination costs with 1 outbreak in 20 years	4%	[15]
**17**	Smaller neonatology ward (HCW × 0,50 and ward occupation ×0,50)	0,50	*Assumed*
**18**	Bigger neonatology ward (HCW × 1,50 and ward occupation ×1,50)	1,50	*Assumed*
**19**	Length of patient restriction uptake on the neonatology ward (5 days) and average length of stay of neonates in neonatology ward (14 days)	5 14	*Assumed*

## Results

Total nosocomial pertussis outbreak costs in the AMC in the Netherlands were €48,682. Direct control cost account for less than 25%. The majority of the costs were caused due to patient uptake restrictions on the neonatology ward, including savings due to reduce working force (33%), and due to absenteeism of HCWs (43%). Medical consumption costs were €785, laboratory investigation costs accounted for €6,982, outbreak management control costs were €3,697, costs due to absenteeism were €21,008, and costs due to patient uptake restrictions were €16,210). Cumulative vaccination costs, including boostering, were €12,208. The return on investment of vaccinating HCWs was 1:4, meaning 4 Euros can be saved by investing one Euro in vaccinating HCWs to prevent a nosocomial pertussis outbreak (Table 3).

**Table 3:** **Return on investment of preventively vaccinating healthcare workers against pertussis**

	**Costs in Euros**	***%***
**Direct control costs**		
Antibiotic therapy *(a)*	€785	*2%*
Laboratory investigations *(b)*	€6,982	*14%*
Outbreak control management *(c)*	€3,697	*8%*
***Total (a,b,c)***	***€11,464***	***24%***
**Other outbreak related costs**		
Absenteeism costs *(d)*	€21,008	*43%*
Restrictions on patient uptake on the ward *(e)*	€47,036	*33%* ^*2*^
Savings due to reduced staff costs *(f)* ^*1*^	−/− €30,826	
***Total (d,e,f)***	***€37,218***	***76%***
***Total nosocomial pertussis outbreak costs (g)***	***€48,682***	***100%***
**Vaccination costs** ***(h)***	€12,208	
	**Ratio**	
***Return on investment ((g-h)/h)***	***1:4***	

^*1*^ *Reduced work force was required due to ward closure, which led to savings in personnel costs.*

^*2*^ *(€47,036 + €-30,826)/€48,682 = 33%.*

### Sensitivity analysis

**Figure 1:**
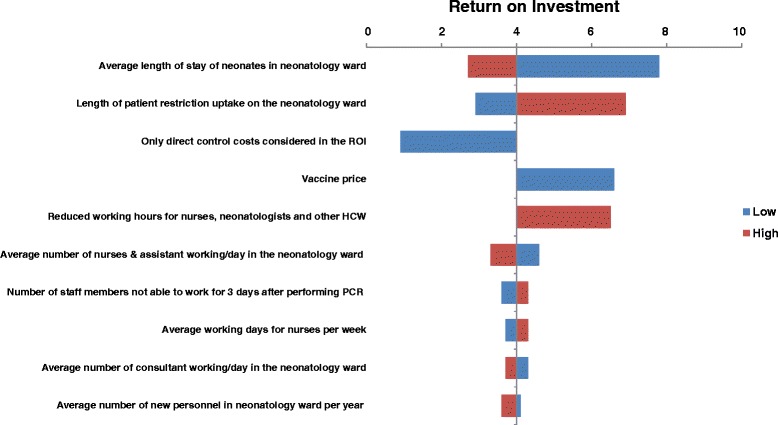
**Tornado diagram with outcomes of the univariate and two-way sensitivity analysis.**

Vaccine price, inclusion of direct control costs only, average length of stay of neonates on the neonatology ward, length of patient uptake restrictions, assuming no reduced work force due to ward closer, and presuming two outbreaks would occur in 10 years time had an impact on the ROI, see Figure 1 (for detailed information see Table A.1 in Additional file 2). The ROI increased to 1:6.6 when vaccine price was decreased to €18.30 per dose. When only direct control costs were considered in the ratio, ROI was slightly negative (1:-0.9). The ROI was 1:7.8 when average length of stay of neonates in the ward was assumed to be shorter (i.e. 7 days versus 14 days), and would be 1:2.7 if average length of stay of neonates would be 21 days. A shorter and a prolonged length of patient uptake restrictions resulted in a lower (1:2.9) and a higher (1:6.9) ROI, respectively. Assuming no reduction in the work force on the neonatology ward resulted in a ROI of 1:6.5. Presuming an outbreak would occur twice in 10 years, the ROI would be 1:7.9, if undiscounted and 1:7.4 if discounted. All other factors, including discounting, changed only slightly the calculated ROI.

## Discussion

The return on investment of preventively vaccinating HCWs against pertussis to prevent a nosocomial pertussis outbreak was 1:4, meaning 4 Euros can be saved by investing one Euro on preventive vaccination of HCWs to prevent a pertussis outbreak. Total nosocomial pertussis outbreak costs in the AMC were €48,682. Direct control costs and other outbreak related costs were 24% and 76% of total costs, respectively. The majority of the costs were caused due to patient uptake restrictions on the neonatology ward of the hospital and by absence of the infected HCWs.

Our findings on total outbreak costs were in accordance with Ward et al., [5] (€55,579) and Calugar et al., [1] (€76,945). However, costs reported by Baggett et al., [8] (€114,526 and €248,998) were much higher compared to our study, which was primarily the result of higher personnel costs used in Baggett et al., [8]. Our estimate of the ROI was slightly higher than calculated by Calugar et al., [1] but still in the same order of magnitude.

### Limitations & assumptions

A major limitation of this study is the possibility of recall bias because the data on the pertussis outbreak were recalled from the year 2004. Another limitation is the narrow perspective (i.e. hospital ward) used in this study. However, using a broader perspective and including additional costs would have led to even a more favourable (i.e. higher) ROI. The assumptions made in this study led to outbreak cost estimates which can be considered as conservative. First, handling costs of several activities (e.g. drug and vaccination administration, PCRs, blood samples, and survey) were not considered as it was assumed that these activities were performed by the staff themselves during their regular working hours. Including the costs of these activities would lead to higher total outbreak costs and a higher ROI. Second, it was assumed that no further transmission of the infection took place when patient uptake restrictions on the neonatology ward were lifted. In practice, additional infections could occur after these restrictions would be lifted, which would lead to additional outbreak costs and a higher ROI. Third, additional costs related to the spread of the infection by children who were brought to other hospitals were beyond our perspective (i.e. the hospital ward) and therefore not considered. But also productivity losses due to work absence of sick parents (i.e. only fathers as mothers would be on maternity leave) were disregarded because of the restricted perspective. Both - negative externalities to the Dutch society - might be omitted if the HCWs would have been vaccinated. Fifth, it was assumed that vaccine uptake, both in existing and newly joined HCWs, would be 100%, which is slightly higher than the observed vaccine coverage in general population (i.e. 96-97%) [10]. Also did we assume that a 8-year booster vaccination would be sufficient to guarantee a 100% vaccine effectiveness, which might have been an oversimplification. Unvaccinated and or unprotected individual HCWs, however, remain a risk of infection, and as such a risk for a potential pertussis outbreak. Sixth, psychological impact on parents with newborns due to a prolonged stay and treatment in the hospital was not quantified. Seventh, the prevented outbreak costs were based on one single outbreak. A larger or a smaller outbreak in a slightly other setting might lead to higher or smaller ROI than presented in the current study. Finally, the ROI estimated in this study is based on preventing one nosocomial pertussis outbreak. Actually, the impact of immunization of HCWs may be much larger as pertussis infection occurring in infants might go unrecognized unless extensive lab diagnosis is applied. Every 2–4 years, an extra epidemic is observed with high number of pertussis cases in adolescents and adults in The Netherlands [10]. However, the number of detected nosocomial outbreaks affecting infants does not occur at the same rate which suggests that pertussis infection occurring in infants might go unrecognized. As a consequence, more nosocomial outbreaks could possibly be prevented by preventive vaccination of HCWs, which would lead to a higher ROI. Therefore, the results presented in this study can be considered as conservative.

### Policy implications

In the Netherlands, a national vaccination recommendation of HCWs against pertussis has yet to be made. In the Dutch society, infants are not fully protected against pertussis in the first few months of their life. To provide protection to this vulnerable group, preventive vaccination of HCWs working with vulnerable infants who are not fully protected could be a relevant intervention. Considering the fact that about 76% of the outbreak costs estimated in this study were caused due to patient uptake restrictions on the neonatology ward of the hospital and by absence of the infected HCWs, it shows the importance of preventing nosocomial pertussis outbreaks and their disregarded impact. Also, it could be argued that hospitals as employers should have some responsibility in preventing nosocomial infections and protecting both, patients and staff members. Therefore, within policy decision making on vaccination recommendations, vaccinating HCWs should also be recommended.

## Conclusion

In conclusion, the current study demonstrated that from a hospital ward perspective, preventive vaccination of healthcare workers against pertussis to prevent nosocomial pertussis outbreaks does result in a positive return on investment (1:4). Therefore, preventive vaccination of healthcare workers can be considered a wise use of healthcare resources enabling the prevention of nosocomial pertussis outbreaks with the tendency to reduce, both the economic and disease burden of pertussis in both, hospital setting and the society.

### Ethics approval and consent

As data from the nosocomial pertussis outbreak in the AMC in The Netherlands was used in an aggregated way to model the return on investment of vaccinating healthcare workers without identifying the individual participant, no written informed consent and ethical approval were required from participants to perform the data analysis.

### Standards of reporting

As this study is not a full economic evaluation but a financial analysis based on a mathematical model, the Consolidated Health Economic Evaluation Reporting Standards checklist (CHEERS) was not necessarily suitable to be used in this case. However, while preparing this manuscript the attempt has been to follow where applicable the CHEERS guideline and to meet the reporting standards of a scientific publication.

### Data availability

All relevant raw data used in this study are presented in the current manuscript (i.e. Tables 1, 2 and 3, Figure 1, Box A.1 in Additional file 1 and Table A.1 in Additional file 2) and will be freely available to any scientist wishing to use them for non-commercial purposes, without breaching participant confidentiality.

### Previous publication data

Data from this manuscript has been presented as a poster presentation at the International Society for Pharmaco-economoc and Outcomes Research in Amsterdam in 2014. The abstract of this poster presentation was published in Value of Health Vol. 17, Issue 7, Page A672.

## Endnotes

^1^Cost estimates are shown for 2012 price level using local Consumer Price Indexes: http://stats.oecd.org/Index.aspx?DataSetCode=MEI_PRICES#.

^2^Cost estimates are shown for 2012 price level using exchange rate from USD 2012 to EURO 2012: 0,778 (http://stats.oecd.org/index.aspx?queryid=169).

## Additional files

Additional file 1:
**Box A.1.** Case study (Nosocomial pertussis outbreak on the neonatology ward in the AMC in the Netherlands in 2004). Additional file 2:
**Table A.1.** Applied one-way and two-way sensitivity analyses: details and results. Costs are expressed in 2012 Euros.

## Abbreviations

AMC: Academic Medical Center Amsterdam
CPI: Consumer price index
HCWs: Healthcare workers
IPV: Inactivated polio vaccine
PCR: Polymerase chain reaction test
ROI: Return on investment
USD: United States of America Dollar
