# Supplementary material for: Modelling the return on investment of preventively vaccinating healthcare workers against pertussis
Source: BMC Infect Dis. 2015 Feb 19;15:75. doi: 10.1186/s12879-015-0800-8 (PMC4340637; doi:10.1186/s12879-015-0800-8)
Supplement: Additional file 1: — Box A.1. Case study (Nosocomial pertussis outbreak on the neonatology ward in the AMC in the Netherlands in 2004). [file 12879_2015_800_MOESM1_ESM.docx]

**Box A.1: Case study**

**Nosocomial pertussis outbreak on the neonatology ward in the AMC in the Netherlands in 2004**

On June 11, 2004 a secretary of the neonatology ward of the AMC experienced cold symptoms with cough. From June 28 onwards, this staff member was on sick-leave. On July 6, the occupational health service performed serological tests for pertussis, which resulted in high antibodies of *Bordetella pertussis*, caused by a recent pertussis infection. Two nurses and a physician working on the neonatology ward were infected. Laboratory investigations reported additional five infected cases. In total, nine pertussis cases were reported. Several outbreak-containment measures were undertaken in the hospital related to patients, their parents, and staff members of the neonatology ward.

Patients and parents

All newborns (n=20) were tested on pertussis with Polymerase Chain Reaction tests (PCR) on nasopharyngeal swabs and blood samples for serology were taken. All newborns received erythromycin during 14 days. Their parents (n=40) were treated with azithromycin during three days (i.e. fathers (n=20) and non-lactating mothers (n=7)) or erythromycin during 14 days (i.e. lactating mothers (n=13)). There were also newborns transferred to another hospital (n=23). Their parents were informed (telephone calls) about their child being at-risk of developing pertussis.

Staff members

Of the 133 staff members, including consultants, domestic cleaners, and all other persons who visited the ward at least once during the outbreak period most received azithromycin during three days. Erythromycin during 14 days was only given to lactating and pregnant staff members (n=5). Only one staff member refused to receive prophylaxis and wore a surgical mask for three weeks. Nasopharyngeal swabs for PCR (n=24) and blood samples for serology (n=27) were taken.

Other measures

In the hospital, crisis meetings (n=5) were organized during the outbreak period with ten HCW present at every meeting. In addition, one neonatologist spent one whole week working on controlling the outbreak. Furthermore, a survey was performed by the occupational health service department to investigate the experience of all staff members (n=133) on receiving prophylaxis. Also, there was a restriction on the intake of new patients on the neonatology ward for ten days.

For more information on the nosocomial pertussis outbreak in the AMC in The Netherlands, please see Zwart et al., [11].
