# Supplementary material for: Modelling the return on investment of preventively vaccinating healthcare workers against pertussis
Source: BMC Infect Dis. 2015 Feb 19;15:75. doi: 10.1186/s12879-015-0800-8 (PMC4340637; doi:10.1186/s12879-015-0800-8)
Supplement: Additional file 2: — Table A.1. Applied one-way and two-way sensitivity analyses: details and results. Costs are expressed in 2012 Euros. [file 12879_2015_800_MOESM2_ESM.docx]

| **Nr.** | **Scenarios** | **Value** | **Total outbreak costs** | **Vaccination costs** | **ROI** |
| --- | --- | --- | --- | --- | --- |
| **1** | **Base case** |  | **€48.682** | **€12.208** | **4,0** |
| **2** | Average working days for nurses per week (Base: 4 days) | 3 | €44.973 | €12.208 | **3,7** |
|  |  | 5 | €52.391 | €12.208 | **4,3** |
| **3** | Average length of stay of neonates in neonatology ward (Base: 14 days) | 7 | €95.718 | €12.208 | **7,8** |
|  |  | 21 | €33.004 | €12.208 | **2,7** |
| **4** | Length of patient restriction uptake on the neonatology ward (in days) (Base: 10 days) | 5 | €35.025 | €12.208 | **2,9** |
|  |  | 15 | €83.719 | €12.208 | **6,9** |
| **5** | Average number of nurses & assistant working/day in the neonatology ward (Base: 30) | 20 | €56.456 | €12.208 | **4,6** |
|  |  | 40 | €40.909 | €12.208 | **3,3** |
| **6** | Average number of consultant working/day in the neonatology ward (Base: 10) | 5 | €52.435 | €12.208 | **4,3** |
|  |  | 15 | €44.930 | €12.208 | **3,7** |
| **7** | Number of staff members not able to work for 3 days after performing the PCR test | 0 | €44.240 | €12.208 | **3,6** |
|  |  | 10 | €53.124 | €12.208 | **4,3** |
| **8** | Average number of new personnel in neonatology ward /year (in %) (Base: 10%) | 5 | €48.682 | €11.772 | **4,1** |
|  |  | 15 | €48.682 | €13.625 | **3,6** |
| **9** | No reduced working hours for nurses, neonatologists and other HCW due to ward closure | 0 | €79.508 | €12.208 | **6,5** |
| **10** | Vaccine price | €18,30 | €48.682 | €7.382 | **6,6** |
| **11** | Costs considered in the ROI - only direct control costs | €11.464 | €11.464 | €12.208 | **0,9** |
| **12** | Undiscounted outbreak and vaccination costs with 2 outbreaks in 10 years | 0% | €97.364 | €12.208 | **7,9** |
| **13** | Discounted outbreak and vaccination costs with 1 outbreak on year 5 in 10 years | 4% | €40.013 | €11.059 | **3,6** |
| **14** | Discounted outbreak and vaccination costs with 2 outbreaks (on year 2.5 and 7.5) in 10 years | 4% | €82.004 | €11.059 | **7,4** |
| **15** | Undiscounted outbreak and vaccination costs with 1 outbreak in 20 years | 0% | € 48.682 | € 18.748 | **2,6** |
| **16** | Discounted outbreak and vaccination costs with 1 outbreak in 20 years | 4% | € 22.218 | € 14.401 | **1,5** |
| **17** | Smaller neonatology ward (HCW x 0,50 and ward occupation x0,50) |  | € 25.059 | €6.104 | **4,1** |
| **18** | Bigger neonatology ward (HCW x 1,50 and ward occupation x1,50) |  | € 72.306 | € 18.312 | **3,9** |
| **19** | Length of patient restriction uptake on the neonatology ward (5 days) and Average length of stay of neonates in neonatology ward (14 days) |  | € 35.025 | € 12.208 | **2,9** |
